# Test Re-test Reliability of Single and Multijoint Strength Properties in Female Australian Footballers

**DOI:** 10.1186/s40798-020-00292-5

**Published:** 2021-01-09

**Authors:** Daniel Kadlec, Matthew J. Jordan, Leanne Snyder, Jacqueline Alderson, Sophia Nimphius

**Affiliations:** 1 School of Medical and Health Sciences, Edith Cowan University, 270 Joondalup Drive, Joondalup, WA 6027 Australia; 2 Canadian Sport Institute Calgary, ABDISO, Calgary, T2N 1N4 Canada; 3 School of Human Sciences, The University of Western Australia, Perth, Australia; 4 Sports Performance Research Institute New Zealand (SPRINZ), Auckland University of Technology, Auckland, New Zealand

## Abstract

**Purpose:**

To examine the test re-test reliability of isometric maximal voluntary contractions (MVC) of hip adduction (ADD_ISO_), hip abduction (ABD_ISO_), and multijoint leg extension (SQUAT_ISO_) in sub-elite female Australian footballers.

**Methods:**

Data were collected from 24 sub-elite female Australian footballers (age 22.6 ± 4.5 years; height 169.4 ± 5.5 cm; body mass 66.6 ± 8.0 kg; 4.5 ± 4.4 years sport-specific training; 2.5 ± 2.0 years unstructured resistance training) from the same club on two non-consecutive days. Participants performed three isometric MVCs of ADD_ISO_, ABD_ISO_, and SQUAT_ISO_. The SQUAT_ISO_ was performed at 140° knee flexion with a vertical trunk position and ADD_ISO_ and ABD_ISO_ measures were performed in a supine position at 60° of knee flexion and 60° hip flexion. Reliability was assessed using paired *t* tests and the intraclass correlation coefficient (ICC) with 95% confidence intervals (CI), typical error (TE), and coefficient of variation (CV%) with 95% CI.

**Results:**

SQUAT_ISO_ peak force (ICC .95; CV% 4.1), ABD_ISO_ for left, right, and sum (ICC .90–.92; CV% 5.0–5.7), and ADD_ISO_ for left, right, and sum (ICC .86–.91; CV% 6.2–6.9) were deemed acceptably reliable based on predetermined criteria (ICC ≥ .8 and CV% ≤ 10).

**Conclusion:**

SQUAT_ISO_, ABD_ISO_, and ADD_ISO_ tests demonstrated acceptable reliability for the assessment of peak force in sub-elite female Australian footballers, suggesting these assessments are suitable for muscle strength testing and monitoring adaptations to training.

**Supplementary Information:**

The online version contains supplementary material available at 10.1186/s40798-020-00292-5.

## Key Points

- Hip abduction, hip adduction and multijoint isometric strength testing demonstrated high test-retest reliability in sub-elite female athletes with limited structured resistance training experience.
- The assessments examined in the current study may be a valuable component of athlete monitoring for readiness and fatigue and as a component of a comprehensive test battery for assessing injury risk
- The results from the current study can contribute to the development of normative data for isometric ADD_ISO_, ABD_ISO_, and SQUAT_ISO_ strength in sub-elite female athletes.

## Introduction

Lower limb maximal muscle strength is important for athletic performance and injury prevention [1] and is often assessed and monitored in an athletic population [2]. Multijoint isometric strength testing such as the isometric mid-thigh pull or isometric squat (SQUAT_ISO_) demonstrate good test-retest reliability and may involve a lower injury risk compared with repetition maximum (RM) strength testing using free weights. The reliability of multijoint isometric strength testing has been investigated in elite female athletes who are experienced with strength training [3]; however, studies investigating the reliability of SQUAT_ISO_ with sub-elite female athletes with limited structured resistance training experience are lacking. Further, while multijoint testing has been shown to be reliable, it is possible that gross motor strategy used during multijoint testing may lack sensitivity to identifying isolated fatigue [4] and therefore it may be useful to further evaluate the reliability of multijoint and single-joint assessments within the same cohort.

In addition to multijoint isometric strength testing, single joint isometric strength assessments are often used in athletic populations to assess lower limb maximal strength due to the relationship between isolated joint strength, lower limb injuries, and performance. For example, athletes with groin pain have been shown to demonstrate lower hip adduction (ADD_ISO_) maximal strength compared to healthy athletes [5]. Also, a positive association has been demonstrated between decreased hip abduction (ABD_ISO_) strength and lower limb movement mechanics associated with injury [6] alongside an increased generalized risk for knee injury [7]. However, ABD_ISO_ and ADD_ISO_ strength testing reliability has not been reported in sub-elite female Australian football. The importance of reliable hip strength measurement to assist in identification of lower limb injury risk is of clear importance considering the known priority to consider risk of ligamentous knee injuries in this population [8, 9].

The purpose of this study was to examine the test-retest reliability of isometric maximum voluntary contractions (MVCs) of ADD_ISO_, ABD_ISO_, and SQUAT_ISO_ in sub-elite trained female Australian footballers. We hypothesized that the test-retest reliability conducted with sub-elite trained female athletes would be acceptable and comparable to that reported for elite athletes.

## Methods

### Participants

Female sub-elite Australian footballers (*n* = 24; age 22.6 ± 4.5 years; height 169.4 ± 5.5 cm; weight 66.6 ± 8.0; 4.5 ± 4.4 years sport-specific training; 2.5 ± 2.0 years resistance training) were recruited from a single club from two competition grades—West Australian Football League Women (WAFLW) and WAFLW Reserves squad. Participants indicated they had no previous history of engaging in supervised structured resistance training program despite reported resistance training of free/unstructured resistance training. All participants provided written informed consent prior to testing (University Research Ethics Approval #22459). Isometric MVCs were obtained from two non-consecutive testing sessions separated by 48 h. Participants performed a standardized warm up prior to the isometric MVCs consisting of five bodyweight squats, five backwards lunges per side, and five submaximal countermovement jumps. The testing sessions were supervised by the same qualified exercise testing practitioner who instructed the participants to push as “fast and as hard as possible” and provided strong verbal encouragement throughout the test.

### Isometric Squat

Participants performed the SQUAT_ISO_ against a fixed barbell in a squat rack at a joint angle of 140° knee flexion while maintaining an upright trunk position determined using a hand-held goniometer. Foot-width was measured and remained consistent between trials and testing days. Participants then performed warm-up contractions including a 5-s submaximal contraction with 50% effort followed by a 3-s contraction at 70–80% effort prior to performing the isometric MVCs. Participants completed three 5-s isometric MVCs separated by a 2-min rest period [10]. The vertical ground reaction force was obtained from each limb using a dual force plate system (PASPO Force Platform PS-2141, PASCO, Roseville, USA) that sampled at 1000 Hz. The trial with the highest maximum force was used in the statistical analysis.

### Isometric Hip Strength

Participants were positioned beneath a Force Frame Strength Testing System (Vald Performance, Albion, Australia) in a supine position with 60°of knee flexion and the feet placed flat on the ground. The femoral condyles were positioned in the center of the dynamometer and force was recorded from each limb simultaneously using load cells sampling at 50 Hz. Participants performed a 5-s submaximal contraction with 50% effort followed by a 3-s contraction at 70–80% effort prior to performing the isometric MVCs. Participants then completed three 5-s isometric MVCs of ABD_ISO_ and ADD_ISO_ separated by a 30-s rest period. The maximum left and right isometric force and maximum total isometric force (sum of left and right) were obtained, and the trial with the maximum total force was used for analysis.

### Statistical Analysis

Mean, standard deviation (SD), and 95% confidence intervals [CI] were calculated for each variable. Paired samples *t* test with significance set at *p* < .05 and intraclass correlation coefficient 3,1 (ICC) were calculated in R (R Core Team 2018, http://www.R-project.org/; see supplemental code for packages). In addition, typical error (TE) and coefficient of variation (CV) of the log-transformed data with 95% CI was calculated by published formula (https://www.sportsci.org/resource/stats/xrely.xls). Acceptable reliability was established by predetermined criteria of an ICC ≥ .8 and CV ≤ 10% based on interpretation of previous recommendations [11].

## Results

**Fig. 1:**
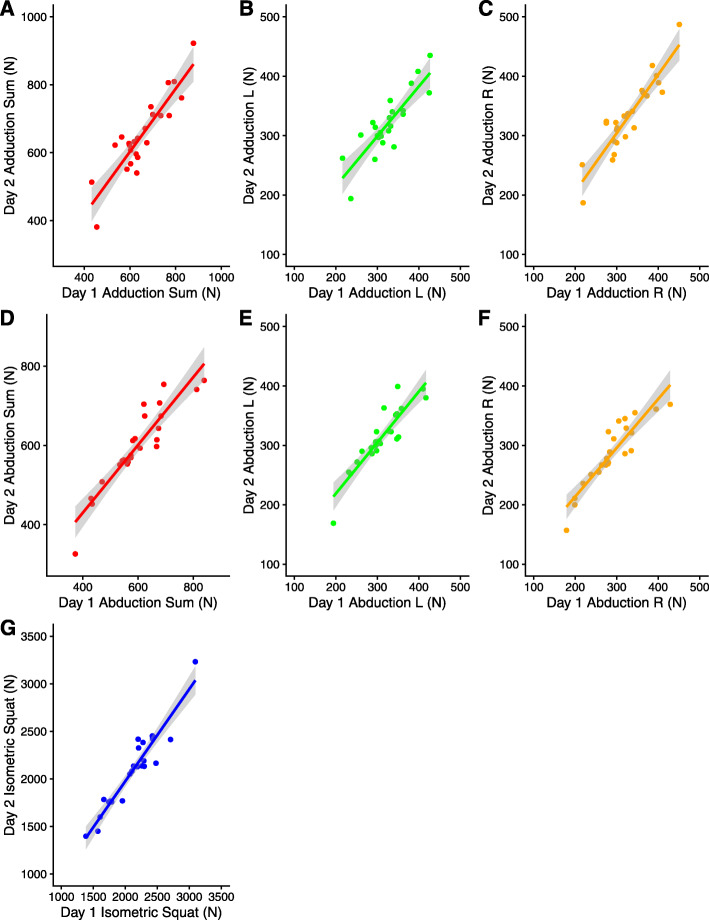
Test re-test data visualized for each isometric assessment. **a** Adduction sum of left and right. **b** Left adduction. **c** Right adduction. **d** Abduction summed from left and right. **e** Left abduction. **f** Right adduction. **g** Isometric squat. Data is presented with a linear fit and 95% confidence cloud for visual observation of all data points

No significant differences between testing session one and testing session two were observed for SQUAT_ISO_ or ADD_ISO_ and ABD_ISO_ for left, right, or sum (Table 1) supportive of absolute reliability. All isometric tests assessed demonstrated acceptable relative reliability as detailed in Table 1 with the range for ICC of .86–.95 and CV% of 4.1–6.9. Data appeared normally distributed and followed a linear pattern (Fig. 1) supporting assessment of relative and absolute reliability assessments reported in Table 1.

**Table 1:** Test re-test reliability of the isometric squat, abduction and adduction maximal force. Session scores expressed as mean (SD)

	Day 1	Day 2	*p*	ICC_3,1_ [95% CI]	TE	CV % [95% CI]
SQUAT_ISO_ (N)	2127 (393)	2099 (401)	.30	.95 [.88–.98]	91	4.1 [3.2–5.8]
ADD_ISO_ sum (N)	651 (108)	650 (112)	.90	.90 [.78–.95]	36	6.4 [4.9–9.0]
ADD_ISO_ left (N)	323 (52)	319 (51)	.39	.86 [.70–.94]	19	6.9 [5.4–9.9]
ADD_ISO_ right (N)	327 (58)	330 (62)	.48	.91 [.81–.90]	18	6.2 [4.8–8.8]
ABD_ISO_ sum (N)	598 (110)	599 (102)	.87	.92 [.83–.97]	30	5.0 [3.9–7.1]
ABD_ISO_ left (N)	310 (53)	313 (51)	.49	.90 [.79–96]	17	5.5 [4.2–7.7]
ABD_ISO_ right (N)	287 (59)	285 (52)	.70	.91 [.80–96]	17	5.7 [4.4–8.1]

*SQUAT*_*ISO*_ isometric squat, *ADD*_*ISO*_ adduction, *ABD*_*ISO*_ abduction, summated left and right ADD and ABD are represented by “sum”, *ICC*_*3,1*_ intraclass-correlation coefficient, *95% CI* 95% confidence interval, *TE* typical error, *CV* coefficient of variation. Significance set at *p* < .05

## Discussion

Consistent with previous research and with the study hypothesis, ADD_ISO_, ABD_ISO_, and SQUAT_ISO_ testing demonstrated high test-retest reliability in a sub-elite female athlete population (Table 1; Fig. 1). The test-retest reliability of the SQUAT_ISO_ test employed in the present study (ICC .95 [.88–.98]; CV% 4.1 [3.2–5.8]), was similar to that reported in male and female elite athletes (ICC .97 [.94–.99]; CV% 4.6) [3], evidencing the utility of the SQUAT_ISO_ test for monitoring lower limb maximal muscle strength in sub-elite female athletes. Absolute SQUAT_ISO_ strength in the current study (day 1 2127 ± 393 N; day 2 2099 ± 401 N) were similar to previously reported strength female athletes with at least 6 months of resistance training experience (2090 ± 578 N) [3]. The combined results provide support for normative strength values for female athletes and can help practitioners establish standards and identify strength deficits. Consistent with this finding, the reliability of the ABD_ISO_ found in the present study (sum ABD_ISO_: ICC .90; CV% 6.4) was comparable to the reliability measures reported in a cohort of professional male Australian footballers using the same testing device (ABD_ISO_: ICC .94; CV% 6.3) [10]. Further, higher reliability was found for the ADD_ISO_ and ABD_ISO_ tests (ICC .86–.92; CV% 5.0–6.9) used in this investigation compared with adduction strength assessed using a sphygmomanometer (ICC .86; CV% 7.6) [12] and isometric hip abduction strength assessment using a hand-held dynamometer (ICC .81–.84) [13].

The present findings are relevant to sport science, sport medicine, and sport performance practitioners as lower body maximal muscle strength including hip abduction and adduction strength are important for performance and injury risk identification in female athletes [1]. Importantly, the testing sessions conducted here were not preceded by familiarization trials and measurements were obtained from female athletes with minimal resistance training experience. As injuries associated with lower limb strength imbalances affect elite and sub-elite athletes alike, reliable measures of multijoint and single joint muscle strength are essential [5, 8]. In addition to evaluating lower limb muscle strength for injury risk purposes, the reliability of the methods assessed in this study suggest that these testing methods are well suited for routine athlete monitoring practices aimed at examining changes in lower body muscle strength.

## Conclusion

Consistent with the hypothesis, the present investigation demonstrated high reliability for ABD_ISO_, ADD_ISO_, and SQUAT_ISO_ testing in sub-elite trained female Australian rules footballers with limited resistance training experience. The results from the current study can contribute to the development of normative data for isometric ADD_ISO_, ABD_ISO_, and SQUAT_ISO_ strength in sub-elite female athletes. Therefore, the assessments examined in the current study may be a valuable component of athlete monitoring for readiness and fatigue and as a component of a comprehensive test battery for assessing injury risk.

## Additional Files

**Additional file 1:.** Supplementary Data. De-identified data for test re-test of all participants.

**Additional file 2:.** Analysis Code. R Script for data analysis.

## Data Availability

All data generated or analyzed during this study are included in this published article [and its supplementary information files].

## Abbreviations

ABD_ISO_: Isometric hip abduction
ADD_ISO_: Isometric hip adduction
ACL: Anterior cruciate ligament
CI: Confidence intervals
CV%: Coefficient of variation
ICC: Intraclass correlation coefficient
MVC: Maximal voluntary contractions
RM: Repetition maximum
SQUAT_ISO_: Isometric squat
TE: Typical error
